# Global analysis of overweight prevalence by level of human development

**DOI:** 10.7189/jogh.05.020413

**Published:** 2015-12

**Authors:** Carmen D Ng

**Affiliations:** University of Pennsylvania, Philadelphia PA, USA

## Abstract

**Background:**

Less developed countries are increasingly afflicted with over–nutrition, and the escalating overweight prevalence has become a global problem. However, a problem as global as this may not be amenable to a general set of remedial interventions applicable to all countries.

**Methods:**

I use data from various sources, including the World Health Organization and the World Bank, to test the association of overweight prevalence with economic, social, and demographic indicators. I then split the countries up by human development index to investigate to what extent these associations vary between development levels.

**Findings:**

On a global scale, overweight prevalence is most associated with gross domestic product (GDP) per capita, the proportion of a country that is rural, the proportion of elderly in a country's population, and the average years of schooling. At what magnitude, and even in which direction, these relationships go vary with a country's level of development. Generally, GDP per capita has a positive association with overweight prevalence, with the magnitude of such association for countries of very high human development more than twice of that for countries of low human development. However, proportion rural has a negative association with overweight prevalence, with the magnitude of such association for countries of low human development nearly twice of that for countries of very high human development. All four of these variables have statistically significant association with overweight prevalence in countries with low human development.

**Conclusions:**

I make policy suggestions to combat increasing overweight prevalence, based on the models that are developed, paying special attention to the differences in magnitude and direction of the regressors between human development levels.

According to World Health Organization (WHO) statistics, more than a billion adults are overweight. WHO defines adult overweight as having a body mass index (BMI) of 25 kg/m^2^ or above. The rise of overweight prevalence has been an issue in developed countries for years, but it has gained increasing attention in developing countries as an issue that needs to be addressed. Traditionally, being underweight has been a “poor country’s problem,” whereas being overweight has been a “rich country’s problem.” Now, developing countries are plagued with both [1].

The rapid increase in economic development, urbanization, and industrialization has been a major reason for the rise in overweight prevalence in developing countries. This transformation has led to substantial changes in diet and physical activity, an increasing prevalence of being overweight, and accompanying conditions and diseases. Studying overweight prevalence is important because it is a risk factor for many non–communicable diseases, including cardiovascular disease, diabetes, and certain types of cancer [2].

Much research has been conducted on overweight prevalence and accompanying policy recommendations [3,4]. However, a problem as global as this may not be amenable to a general set of remedial interventions applicable to all countries. The difference between developed and developing countries in terms of economic, social, and demographic indicators is so tremendous that the same policies could not be expected to work for both. The purpose of this paper is to analyze adult overweight prevalence in countries at varying degrees of development, measured by such aggregate indicators. I expect the associations of these factors with overweight prevalence to vary between countries at different levels of development, so a one–size–fits–all policy would not work for all countries. I seek to interpret these results, and suggest actions that countries could take, depending on their development level.

## METHODS

In this study, a macro approach of investigation is taken, using country–level data from 2002, 2005, and 2010. I choose the above years based on the availability of data from the WHO Global Infobase, a database with information on chronic diseases and their risk factors [5]. The dependent variable is the percentage of overweight adults aged 15 to 100 in a country.

A multitude of variables potentially related to overweight prevalence are considered. Data for these variables are mainly from the World Bank [6]. A data set with information on the number of McDonald’s restaurants in various countries, compiled by the Datablog of *The Guardian* newspaper, is also used [7]. Variables in my analysis include the following: gross domestic product (GDP) per capita in 2010 US$ and adjusted by purchasing power parity, unemployment rate, percentage of population rural, percentage of population aged 65+, average years of total schooling (ages 15+), internet users (per 100 people), and coverage of McDonald’s restaurants. The variables GDP per capita, proportion of people aged 65+, and internet users exhibit a non–linear concave pattern when plotted with overweight prevalence, so a natural logarithmic transformation (with an offset) is used to linearize the data.

I use the variable internet users as a proxy for sedentary lifestyles and access to Western culture, and the variable coverage of McDonald’s restaurants as a proxy for access to fast food. Coverage is defined as the area of a country (in km^2^) divided by the number of McDonald’s restaurants in the country. For countries that had no McDonald’s restaurants, I give them a value of 350 000, which is greater than any of the values for countries with at least one McDonald’s restaurant. It is reasonable to assume that if a McDonald’s restaurant was not within 350 000 km^2^, a person would not elect to go there. Data for the number of McDonald’s restaurants are available for the years 2007 and 2012 (used for my 2005 and 2010 analyses respectively).

To start, I run multiple linear regression models to investigate the association of overweight prevalence with my explanatory variables among all countries. All analyses are run using the statistical package R (version 3.1.1) [8] and a significance level of five percent is used. With overweight prevalence data for multiple years, I further investigate using a panel model. In addition, I would like to take into account the heterogeneity among countries. The Hausman specification test suggests a fixed effects model. For this model, both sexes and the significant variables from the previous models are included.

The Human Development Index (HDI) is a loose indicator of a country’s level of development complied by the United Nations Development Program (UNDP). The indicator is determined using three factors–life expectancy, an aggregate measure based on the mean years of schooling for adults and the expected years of schooling for children, and gross national income per capita. The HDI is calculated for most countries, and each country is put into one of four categories – very high (1), high (2), medium (3), or low (4) human development [9]. It should be noted that there are potential issues here with reverse causality. For the rest of the paper, HDI level is used only as a means to separate countries into delineated groups.

One–way analysis of variance (ANOVA) and pairwise testing are used to compare mean overweight prevalence between HDI levels. To ascertain the differences in more detail, I construct fixed effects models for the four HDI levels, and compare the difference in association of overweight prevalence with my explanatory variables between pairs of HDI levels.

## RESULTS

There are 192 countries with data on overweight prevalence in 2002, 2005, and 2010. Of these countries, 47 are in the very high, 52 are in the high, 41 are in the medium, 43 are in the low development level, and 9 are not placed into any of these categories as of 2013. My analyses are all run with a subset of these countries, depending on the availability of data for each variable.

### Multiple regression models by sex and year

I run a multiple regression model for each sex and year combination, and the results are presented in **Table 1** and **Table 2**.

**Table 1:** Association between male overweight prevalence and economic, social, and demographic regressors using a multiple linear regression model in each year†

	2010	2005	2002
Intercept	–11.3 (19.4)	–24.1 (19.2)	–28.3 (18.7)
Log(GDP per capita)	4.71 (2.29)*	6.50 (2.15)**	6.87 (2.11)**
Proportion rural	–0.189 (0.083)*	–0.148 (0.0832)	–0.188 (0.0792)*
Log(proportion 65+)	–0.145 (2.34)	1.46 (2.67)	2.20 (2.70)
Average years of schooling	1.63 (0.741)	1.61 (0.709)*	1.78 (0.718)*
Log(internet users per 100 people)	2.32 (2.51)	–0.150 (2.20)	–1.65 (1.99)
McDonald’s coverage	–9.82 × 10^−7^ (1.02 × 10^−5^)	–6.22 × 10^−6^ (1.05 × 10^−5^)	N/A

N/A – not applicable

†Coefficient estimates are displayed with standard errors in parentheses. ** – significance at 1%, * – significance at 5%.

**Table 2:** Association between female overweight prevalence and economic, social, and demographic regressors using a multiple linear regression model in each year†

	2010	2005	2002
Intercept	31.7 (21.0)	25.4 (20.3)	13.7 (20.1)
Log(GDP per capita)	2.02 (2.47)	2.43 (2.27)	4.03 (2.25)
Unemployment	0.542 (0.236)*	0.724 (0.205)***	0.649 (0.196)**
Proportion rural	–0.236 (0.090)*	–0.169 (0.0884)	–0.193 (0.0855)*
Log(proportion 65+)	–7.84 (2.54)**	–9.13 (2.83)**	–7.70 (2.87)**
Average years of schooling	0.998 (0.804)	0.909 (0.756)	1.01 (0.786)
Log(internet users per 100 people)	3.42 (2.71)	4.37 (2.34)	1.80 (2.19)
McDonald’s coverage	7.78 × 10^−7^ (1.13 × 10^−5^)	–4.46 × 10^−6^ (1.12 × 10^−5^)	N/A

N/A – not applicable

†Coefficient estimates are displayed with standard errors in parentheses. *** – significance at 0.1%, ** – significance at 1%, * – significance at 5%.

For both sexes and all years, log(GDP per capita) and average years of schooling are consistently positively associated with overweight prevalence, whereas proportion rural is consistently negatively associated with overweight prevalence. Associations do not seem to be the same for the two sexes. For example, female overweight prevalence is positively and significantly associated with unemployment, but there appears to be no significant association for males–not even when overweight prevalence is regressed only on unemployment in simple regressions; thus, the multiple regression models for males do not include unemployment.

From the results in **Tables 1 and 2**, the explanatory variables log(internet users) and McDonald’s coverage seem to have little association with overweight prevalence. Sedentary lifestyles and unbalanced diets have often been blamed as major players in the “obesity epidemic” in developed countries [10]. However it seems that neither internet use nor proximity to a McDonald’s restaurant has a significant association with overweight prevalence when both developed and developing countries are considered in this study.

Aside from the intercept which consistently increases and the coefficient of log(GDP per capita) which consistently decreases over the years, the coefficients of the other variables do not reveal a clear pattern. For the most part, coefficients that were significant in 2002 remained that way in the following years.

### Fixed effects model

I include both males and females with the variables from the previous models, except log(internet users) and McDonald’s coverage due to their general lack of significance. The results are presented in **Table 3** (additional models are created to include one or both of the variables log(internet users) and McDonald’s coverage, but these variables are again found to be generally insignificant).

**Table 3:** Association between overweight prevalence and economic, social, and demographic regressors in a fixed effects model (both sexes, all years)†

Log(GDP per capita)	5.74 (0.533)***
Unemployment	0.00457 (0.0426)
Proportion Rural	–0.454 (0.0528)***
Log(Proportion 65+)	2.49 (1.08)*
Average years of schooling	–0.183 (0.145)

†Coefficient estimates are displayed with standard errors in parentheses. *** – significance at 0.1%, * – significance at 5%.

It seems that countries with higher GDP per capita, populations comprised of more elderly people, and more highly–urbanized areas tend to have higher overweight prevalence. Average years of schooling and unemployment are not significant in this model.

Log(proportion 65+) has switched in sign from my models for females in **Table 2**. A possible reason is that the flexibility of a fixed effects coefficient for each sex and country combination allows the coefficients of the regressors more “freedom” to reflect their actual effects. If so, the independent variable log(proportion 65+) seems to have a positive association with overweight prevalence. Previous research on seven middle– and low–income countries has found that overweight prevalence is typically higher for older females, and it would make sense for this result to generalize to countries with older populations [11].

**Figure 1:**
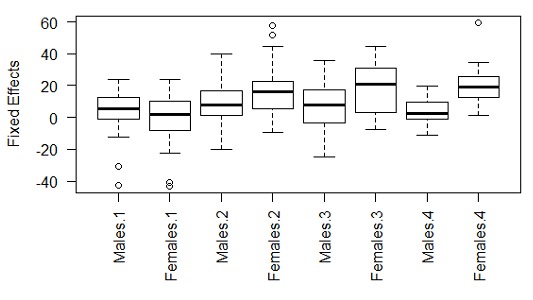
Fixed effects from overweight prevalence fixed effects model. The horizontal axis labels are in the form: sex, Human Development Index (HDI) level (1 for very high, 2 for high, 3 for medium, 4 for low).

**Figure 1** shows boxplots of the fixed effects for each sex and HDI level. If all countries and sexes were homogeneous, in terms of overweight prevalence, I would expect their fixed effects to be about the same. However, that does not appear to be the case. It is interesting that the fixed effects for the females–low human development combination are relatively high even though females in these countries typically have lower overweight prevalence. If the model remains valid, females in countries in the low human development category would be especially at risk of seeing very high overweight prevalence levels as development continues in these countries.

### Analysis by level of development

**Figure 2:**
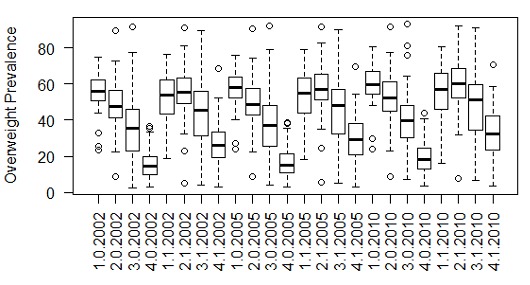
Boxplots of overweight prevalence by HDI level, sex, and year. The horizontal axis labels are in the form: Human Development Index (HDI) level (1 for very high, 2 for high, 3 for medium, 4 for low), indicator for sex (1 for female, 0 for male), and year.

It appears in **Figure 2** that overweight prevalence varies among different levels of development. For each sex and year combination, one–way ANOVA rejects the null hypothesis that the means of overweight prevalence are the same among all four HDI levels. To see which levels have statistically different values of mean overweight prevalence, I perform pairwise t–tests with the Holm–Bonferroni adjustment to correct for multiple testing. For males in all years, the mean overweight prevalence at each HDI level is statistically different from that at every other level. For females in all years, the means of overweight prevalence are statistically different in all pairwise comparisons, other than between very high and high, and between very high and medium. Thus, overweight prevalence in countries seems to vary by their level of development. To delve into this further, I run a fixed effects model for each HDI level. The results of the model are displayed in **Table 4**.

**Table 4:** Association between overweight prevalence and economic, social, and demographic regressors in a fixed effects model by Human Development Index level†

	Very high	High	Medium	Low
Log(GDP per capita)	7.78 (0.883)***	2.84 (1.10)*	8.75 (1.28)***	3.09 (1.19)
Unemployment	0.0405 (0.0594)	–0.0861 (0.0891)	0.00154 (0.125)	–0.160 (0.121)
Proportion rural	–0.423 (0.114)***	–0.203 (0.122)	–0.186 (0.121)	–0.757 (0.0940)***
Log(Proportion 65+)	–0.295 (1.24)	16.1 (4.17)***	4.84 (2.79)	10.9 (3.66) *
Average years of schooling	–0.0467 (0.211)	0.132 (0.340)	0.205 (0.305)	–1.00 (0.311)**

†Coefficient estimates are displayed with the standard errors in parentheses. *** – significance at 0.1%, ** – significance at 1%, * – significance at 5%.

For every pair of HDI levels, I compare the coefficients of each independent variable. To this end, a regression model is created for each pair of HDI levels with added variables for HDI level and interactions of all of my predictors with HDI level. The results of this are shown in **Table 5**. Note that the interaction estimates are consistent with the results in **Table 4**.

**Table 5:** Interactions between economic, social, and demographic regressors and Human Development Index levels from pairwise tests

	Very high vs high	Very high vs medium	Very high vs low	High vs medium	High vs low	Medium vs low
Log(GDP per capita)	–4.94 (1.39)***	0.975 (1.53)	–4.69 (1.50)**	5.92 (1.76)***	0.248 (1.75)	–5.67 (1.75)**
Unemployment	–0.127 (0.103)	–0.0389 (0.135)	–0.201 (0.138)	0.0877 (0.163)	–0.0741 (0.168)	–0.162 (0.175)
Proportion rural	0.220 (0.168)	0.236 (0.165)	–0.334 (0.148)*	0.0166 (0.177)	–0.554 (0.158)***	–0.570 (0.152)***
Log(Proportion 65+)	16.4 (3.88)***	5.14 (2.97)	11.2 (3.97)**	–11.3 (4.95)*	–5.23 (5.80)	6.06 (4.71)
Average years of schooling	0.179 (0.382)	0.252 (0.365)	–0.953* (0.382)	0.0725 (0.465)	–1.13 (0.485)*	–1.20 (0.440)**

†Coefficient estimates are displayed with the standard errors in parentheses. *** – significance at 0.1%, ** – significance at 1%, * – significance at 5%.

The coefficients of unemployment are not significantly different between any pair of HDI levels, nor is this regressor even significant for any HDI level in the fixed effects models. Each pair of HDI levels has at least a couple of variables with significantly different coefficients, with one notable exception. Very high human development and medium human development do not have significantly different coefficients for any variable. It is interesting to note that the means of overweight prevalence are not significantly different between these two levels for females in any year as well. For the most part, the coefficients in the models for the top three HDI levels are pretty different from those for the low human development level.

## DISCUSSION

From the multiple regression models by sex and year, it seems that the associations of overweight prevalence with the economic indicators log(GDP per capita) and unemployment are different for the two sexes, and seem to point in opposite directions. Log(GDP per capita) is consistently positive and significant for males but not for females, while unemployment is consistently positive and significant for females but not for males. This would seem to indicate that males and females tend to be more overweight when the economy is doing well and poorly, respectively. Countries at all levels of development might benefit by offering women with guidance and counseling during economic downturns, and by providing men with reminders to watch their diets and not to forego exercise even while working in a booming economy.

The variable proportion rural is negatively and significantly associated with overweight prevalence in most of my models. In addition, its partial slope in the model for low human development countries is more negative and significantly different from those for other development levels. Previous research among women aged 20 to 49 in a collection of developing countries found that overweight prevalence is about twice as high in urban areas than in rural ones [12]. The negative and significant coefficient of this variable for the very high human development countries is harder to decipher, as research results on urban–rural disparities in these countries have been conflicting [13-15]. Perhaps countries at the very high human development level, ranging from the United States to France to Saudi Arabia, are too heterogeneous. The coefficient of the proportion rural variable is consistently negative, and urbanization decreases such proportion. Countries, especially those with low human development, should make maintaining a generally healthy living environment and establishing quality health care services a priority, as development and urbanization typically occur concurrently.

Log(proportion 65+) is positive and significant in the overall fixed effects model and two of the fixed effects models by HDI. Overweight prevalence appears to be a risk factor for the elderly, and more so for females than males. More assistance for and supervision of elderly females could help reduce overweight prevalence.

Among the four development levels, average years of schooling is significant and negatively associated with overweight prevalence at the low human development level, and this partial slope is significantly different from those in the models for other development levels. For countries at the bottom level of development, education is of the utmost importance. Health education in the least developed countries could help by leaps and bounds in preventing not just overweight prevalence, but also other health conditions. Preventative measures directed toward school children could do more than curative care targeted at already overweight individuals. In addition, more educated parents would likely provide better prenatal and postnatal care, thereby reducing the risk of their children being overweight.

In the individual sex and year models, the coefficient on average years of schooling is always positive and significant for males. How can this sign switch be explained? People with more education are typically wealthier, busier, and have more access to and options for food – all risk factors for increased BMI. In poorer countries though, an additional unit of schooling could substantially contribute to a person’s knowledge of health and/or ability to eat a balanced meal. In a study of reproductive–age women in Egypt, education was found to counter the effects of increasing wealth on overweight prevalence [16].

There are some caveats that should be kept in mind. UNDP switched its method of categorization in 2014 from one based on quartiles to one based on fixed cut–off points. With quartile groupings, a country moving up a quartile necessarily meant that another country would have to move down, even if that country’s level of development had actually improved. However, the fixed cut–offs that have recently been adopted by UNDP might not necessarily work for data from more than a decade ago. Despite their shortcomings, the groupings provided by UNDP in 2013 are used in this study. Countries could have moved between levels and a country’s categorization in 2013 might not be the same as its categorization in 2002, 2005, or 2010, but the UNDP finding that few countries even changed ranks (let alone human development levels) between 2012 and 2013 makes this concern less worrisome [17].

Some of these variables are relatively highly correlated, especially with log(GDP per capita). However, most of the coefficients make sense and are interpretable. Additionally, I check the variance inflation factors to ascertain the severity of this problem for all my regression coefficients in each individual sex and year multiple regression model. All variables have factors well below the criterion indicating a concern.

A few additional comments on the variables are in order. Internet users and McDonald’s coverage are only proxies. They are not stand–ins or perfect numerical representations of sedentary lifestyles and access to Western culture or access to fast food. Additionally, any factors that might be related to overweight prevalence necessarily have a lagging effect. I have not taken such a lag into consideration. Perhaps a one–year lag or a combination of prior values could be used instead in further studies.

It is clear that overweight prevalence is a global problem. However, this is *not* a problem that all countries in the world can address in the same way. Generally, GDP per capita, the proportion of a country that is rural, the proportion of elderly in a country's population, and the average years of schooling are significantly associated with overweight prevalence. Yet the association and significance that each of these variables has on overweight prevalence varies greatly by a country’s level of human development.

I have made some policy recommendations based on my analysis. However, caution is in order. While it seems from the results that increasing GDP per capita would be connected with an increase in overweight prevalence, my conclusion is definitely not to advise policymakers to slow down the development of their countries. Instead, while policymakers try to improve the well–being of their fellow citizens, they should not unwittingly exacerbate the possible ill effects of development, such as increasing overweight prevalence.
